# Placental ABCA1 Expression Is Increased in Spontaneous Preterm Deliveries Compared with Iatrogenic Preterm Deliveries and Term Deliveries

**DOI:** 10.1155/2017/8248094

**Published:** 2017-05-29

**Authors:** Xie Cheng-Mao, Long Yan, Lin Li, Jin Hua, Wang Xiao-Ju, Zhang Jie-Wen

**Affiliations:** Department of Gynecology & Obstetrics, Beijing Friendship Hospital, Capital Medical University, 95 Yong'an Road, Xicheng District, Beijing 100050, China

## Abstract

**Objective:**

Abnormal expression of ABCA1 and ABCG1 in the placenta can elicit lipid metabolism disorder and adverse pregnancy outcomes. However, whether it is associated with preterm delivery remains unclear. Our present study aimed to evaluate the relationship between abnormal expression of ABCA1 or ABCG1 and preterm delivery.

**Methods:**

Maternal blood and placental tissues from women with spontaneous deliveries (SPD), iatrogenic deliveries (IPD), and term deliveries (TD) were collected. The lipid content and expression of ABCA1 and ABCG1 were subsequently measured.

**Results:**

Compared with IPD and TD groups, the HDL, TD, LDL, and TC levels were lower in the maternal blood but higher (except TC) in the cord blood of the SPD group. The extracellular lipid content in the placentas of the SPD group was also notably lower relative to the IPD and TD groups. Moreover, the protein and mRNA expressions of ABCA1 in the placentas of the SPD group were significantly higher compared with the IPD and TD groups; however, there was no obvious difference among the three groups in the protein and mRNA expressions of ABCG1.

**Conclusions:**

Abnormal expression of ABCA1 may be associated with the dysregulation of placental lipid metabolism and the occurrence or development of SPD.

## 1. Introduction

Preterm delivery is defined as delivery between week 28 and week 37 of gestation and may be spontaneous (SPD) or iatrogenic (IPD) [1, 2]. It is the main cause of perinatal morbidity and mortality, as it is associated with more than 75% of perinatal deaths [3]. Moreover, in the long term, the low birth weight of preterm infants presents a significantly higher risk of obesity, hypertension, diabetes, and other metabolic diseases in adulthood [4].

There are a number of risk factors for SPD, which may appear singly or in combination [5, 6]. These risk factors include reproductive tract infection, history of preterm birth, race and genetic factors, age and abnormal fetal position, stress, and socioeconomic factors [7–10]. However, the underlying cause of about 50% of SPD is still largely unknown [11]. Catov et al. [12] have found that high levels of cholesterol and triglycerides in early pregnancy increased the SPD rate 2.8-fold in the first 34 weeks of gestation and twofold in 34–37 weeks. The levels of serum estradiol, progesterone, and corticotropin-releasing hormone in the placenta of pregnant women are also closely linked to the occurrence of SPD [13]. In a preliminary study, we found that women with SPD had lower serum lipid levels and higher umbilical cord blood lipid levels than women with term deliveries (TD) [14]. These findings suggest an association between abnormal lipid metabolism and SPD.

Cholesterol is vital for the development of the human body. In the early stages of embryonic development, cholesterol is required for the maturation of sonic hedgehog protein, which is necessary for morphogenesis [15]. The ATP-binding cassette (ABC) family of transporters has been implicated in various aspects of lipid homeostasis [16, 17]. Two important members of the family, ABCA1 and ABCG1, are known to efflux cholesterol, as well as vitamins, sphingolipids, oxysterols, and phospholipids [16]. ABCA1 and ABCG1 are the main membrane transport proteins responsible for transport of cholesterol to the cells, affecting cellular cholesterol homeostasis. Furthermore, they may play a role in apoptosis and inflammation. Both ABCA1 and ABCG1 are highly expressed in human tissues involved in cholesterol metabolism and steroidogenesis including liver, adrenal glands, testes, and placenta and play a key role in the transfer of cholesterol across the maternal and fetal membranes [18–23].

Maternal cholesterol is the major source of fetal cholesterol [24, 25]. Thus, lipid transport from the mother to the fetus through the placenta is important for fetal development and survival, and fetuses with abnormal cholesterol biosynthesis have a spectrum of developmental abnormalities. Abnormal lipid metabolism is known to cause a number of obstetric complications, including preeclampsia and antiphospholipid syndrome, through its effects on placental cholesterol metabolism and lipid transport [26, 27]. To our knowledge, it is still unknown whether ABCA1 and ABCG1 in the placenta are associated with preterm delivery; hence the objective of our study was to evaluate the relationship between ABCA1 and ABCG1 and preterm delivery.

## 2. Materials and Methods

### 2.1. Subjects

Pregnant women were recruited at Beijing Friendship Hospital from June 2014 to December 2015. Participants were 21–35 years old with a body mass index (BMI) of 19–25 kg/m^2^ and had no intrauterine infection, trauma, history of thromboembolism, severe gastrointestinal tract, heart, lung, or liver disease, chronic hypertension, diabetes, or any other endocrine diseases. Each participant was a healthy primipara and had been confirmed by the ultrasound of a single live fetus. Among these women there were 51 cases of SPD, 46 cases of IPD, and 48 cases of TD.

Placental tissues were collected from near the umbilical cord root area and cut into pieces of uniform size immediately after delivery. One placental sample was used to make paraffin blocks and the other 4-5 samples were stored in liquid nitrogen to preserve the protein and RNA properties. Written consent was obtained from patients prior to the procedure, and the Friendship Hospital Ethics Committee approved all procedures.

### 2.2. Reagents

The following reagents were purchased from commercial sources: mouse monoclonal anti-human ABCA1 antibody (Ab18180) and rabbit monoclonal anti-human ABCG1 antibody (Ab52617; Abcam, Cambridge, UK); mouse monoclonal anti-human *β*-actin antibody (A2228) and Oil Red O (O0625; Sigma Aldrich, St. Louis, MO, USA); ProteoExtract Native Membrane Protein Extraction Kit (M-PEK; Calbiochem, Darmstadt, Germany); Quick Start Bradford protein assay (Bio-Rad Laboratories, Hercules, CA, USA); 7% Tris-acetate gel and TaqMan® Universal PCR Master Mix (Invitrogen, Carlsbad, CA, USA); Mini Protease Inhibitors (Roche, Basel, Switzerland); Immobilon-NC Transfer Membrane (Hybond-C, Amersham International, Little Chalfont, UK); IgG-labeled goat anti-mouse secondary antibody, SP kit, and DAB color reagent (Beijing Zhongshan Golden Bridge Biotechnology Co. Ltd., Beijing, China). The mouse serum, rabbit serum, and all other reagents were from Solarbio (Beijing, China).

### 2.3. Determination of Blood Lipids

Blood lipid levels were determined as previously described [14]. Briefly, we took 3.5 mL of venous blood from each patient just prior to delivery and 3.5 mL of umbilical arterial blood after delivery. Blood samples were sent to the Clinical Laboratory Center in Beijing Friendship Hospital, where total cholesterol (TCHO), high-density lipoprotein (HDL), low-density lipoprotein (LDL), and triglyceride (TG) levels were measured using an AU2700 bioanalyzer (Olympus, Tokyo, Japan).

### 2.4. Determination of Placental Lipids

Oil Red O staining was performed to monitor the progression of adipocyte differentiation, as described previously [25]. Placental tissue sections were fixed in 4% paraformaldehyde and dehydrated in a 30% sucrose solution at room temperature before immersion in optimal cutting temperature solution on dry ice. Blank controls were run using distilled water instead of Oil Red O. The stained fat droplets in the tissues were visualized by light microscopy and photographed. Next, we tested the IOD (integrated option density) values of all the samples with the Image-Pro Plus software (Media Cybernetics, Maryland, USA) for semiquantitative analysis.

### 2.5. Determination of Placental ABCA1 and ABCG1 Expression Levels

#### 2.5.1. Immunohistochemistry

Immunohistochemistry was performed on the formalin-fixed, paraffin-embedded placental samples. Sections cut to 4 *μ*m thickness were heated at 60°C for 10 min, dewaxed, rehydrated, and washed in phosphate-buffered saline (PBS, pH 7.4). They were then incubated in 3% H_2_O_2_ for 10 min to deactivate endogenous peroxidases and washed in PBS 3 × 5 min. Slides were subjected to antigen retrieval in sodium citrate buffer (pH 6.0) in a microwave oven at 850 W for 15 min (3 × 5 min), cooled for 2 h at room temperature, and washed in PBS 3 × 5 min. Samples were preincubated with normal goat serum (1 : 10 in PBS) for 30 min to block nonspecific binding of antibodies, incubated overnight at 4°C with mouse monoclonal anti-human ABCA1 antibody or rabbit monoclonal anti-human ABCG1 antibody (1 : 200 in PBS), and washed in PBS 3 × 5 min. Samples were then incubated with biotinylated goat anti-mouse antibody or mouse anti-rabbit antibody (1 : 500 in PBS) for 30 min at room temperature and washed in PBS 3 × 5 min. Color was developed with DAB and hematoxylin, and the signal was visualized by microscopy. Isotype controls were performed for each sample by substituting the anti-ABCA1 or anti-ABCG1 antibody with mouse serum (ABCA1) or rabbit serum (ABCG1). After that we tested the IOD values of all the samples with the Image-Pro Plus software for semiquantitative analysis.

#### 2.5.2. Protein Preparation and Western Blotting

Western blots were performed on the snap-frozen placental samples. Samples were pulverized under liquid nitrogen and membrane proteins were extracted using the ProteoExtract Native Membrane Protein Extraction Kit according to the manufacturer's instructions. The concentration of the protein extracts was determined using the Quick Start Bradford protein assay. SDS-PAGE was conducted with protein samples of approximately 20 *μ*g loaded onto a 7% Tris-acetate gel, run at 120 V for 2 h. To maintain the integrity of ABCA1 and ABCG1, samples were not heated prior to electrophoresis. After electrophoresis, proteins were transferred onto an Immobilon-NC Transfer Membrane at 300 mA for 90 min. Membranes were blocked in 5% nonfat milk powder in Tris-buffered saline with 0.1% Tween 20 (TBST) for 2 h. They were then incubated with mouse monoclonal anti-human ABCA1 antibody or rabbit monoclonal anti-human ABCG1 antibody (1 : 1000 in TBST) overnight at 4°C and washed with TBST 3 × 10 min. Next, they were incubated with a goat anti-mouse IgG and mouse anti-rabbit IgG-conjugated horseradish peroxidase (HRP) secondary antibody (1 : 5000 in TBST) for 45 min at room temperature and washed in TBST 3 × 10 min. The signal was detected using chemiluminescence (ECL System, Amersham) and *β*-actin was used as an internal control.

#### 2.5.3. RNA Isolation and Quantitative Real-Time Polymerase Chain Reaction (qRT-PCR)

RNA was extracted from snap-frozen placental samples. Samples were removed from liquid nitrogen and placed in precooled TRIzol (Thermo Fisher Scientific, Waltham, MA, USA) reagent. Then, RNA was extracted immediately using chloroform extraction and isopropanol precipitation and quantified using a spectrophotometer. The reverse transcription reaction was carried out according to the manufacturer's instructions in a 20 *μ*L reaction containing 2 *μ*g of total RNA in a gradient cycler (Thermo Fisher Scientific, Waltham, MA, USA). We used successive incubations at 25°C for 10 min and 37°C for 120 min followed by 85°C for 5 min and 4°C for 10 min. LightCycler DNA Master SYBR Green (Roche Diagnostics, Germany) was used to carry out the qRT-PCR for* ABCA1* with the sense primer 5′-GATGGCAATCATGGTCAATGG-3′ and anti-sense primer 5′-AGCTGGTATTGTAGCATGTYCCG-3′ and for* ABCG1* with the sense primer 5′-CAGGAAGATTAGACACTGTGG-3′ and anti-sense primer 5′-GAAAGGGGAATGGAGAGAAGA-3′. The amplicon length was 201 bases, and we used* GAPDH* as an internal control. The PCR reactions contained 3 mM MgCl_2_, 0.4 *μ*M forward and reverse primers, and 1 *μ*L of LightCycler DNA Master SYBR Green I (10x concentrate, Roche). An initial denaturation step of 95°C for 75 s was performed to activate the FastStart DNA Polymerase and to ensure complete denaturation of the cDNA before amplification. Amplification of* ABCA1*,* ABCG1*, and* GAPDH* involved 40 cycles of denaturation at 95°C for 15 s, annealing at 62°C for 10 s, and elongation at 72°C for 25 s. To remove nonspecific signals before SYBR Green I quantification, we added a condition to each amplification cycle involving an elevated temperature fluorescence acquisition point, that is, 80°C for 3 s for* ABCA1* and* ABCG1* and 85°C for 3 s for* GAPDH*. After the last cycle, the amplified products underwent melting curve analysis to check the amplification integrity. Data were analyzed with the Second Derivate Maximum Method using LightCycler Relative Quantification Software.* ABCA1* and* ABCG1* values are expressed relative to* GAPDH*. After the qRT-PCR, we verified the mRNA specificity of* ABCA1* and* ACBG1* with agarose gel electrophoresis.

### 2.6. Statistical Analysis

All the data were analyzed using one-way ANOVA followed by the Student-Newman-Keuls (SNK) method, and differences were considered significant when *P* < 0.05. Values are presented as mean ± SD. SPSS version 17.0 (SPSS Inc., Chicago, IL, USA) was used for all data management and analysis.

## 3. Results

### 3.1. Subject Characteristics and Lipid Levels in Maternal and Cord Blood

**Figure 1:**
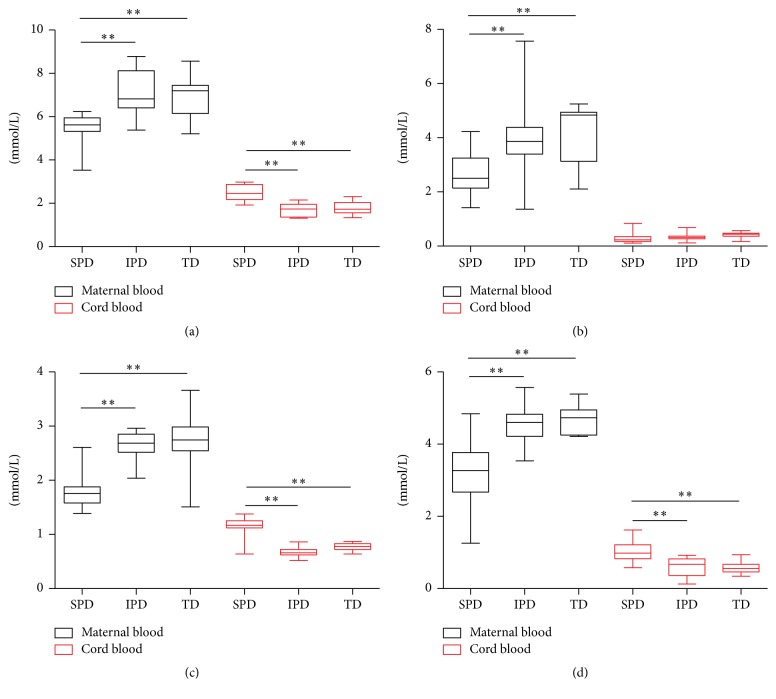
Lipid levels in the maternal blood and cord blood of women with SPD, IPD, or TD. (a) Total cholesterol; (b) triglycerides; (c) high-density lipoprotein; (d) low-density lipoprotein. Statistical significance was determined by one-way ANOVA followed by SNK (^*∗∗*^*P* < 0.01).

The age, BMI, and gestational age at delivery of each subject are presented in Table 1. There were no significant differences in age or BMI among SPD, IPD, and TD. The levels of HDL, TG, LDL, and TCHO in the maternal blood were lower in the SPD than in the IPD and TD, while HDL, LDL, and TCHO (but not TGs) in the cord blood were significantly higher in the SPD than in IPD and TD. There were no differences in HDL, TG, LDL, or TCHO in the maternal or cord blood between the IPD and TD (Figure 1).

### 3.2. Lipid Content in Placental Tissues

**Figure 2:**
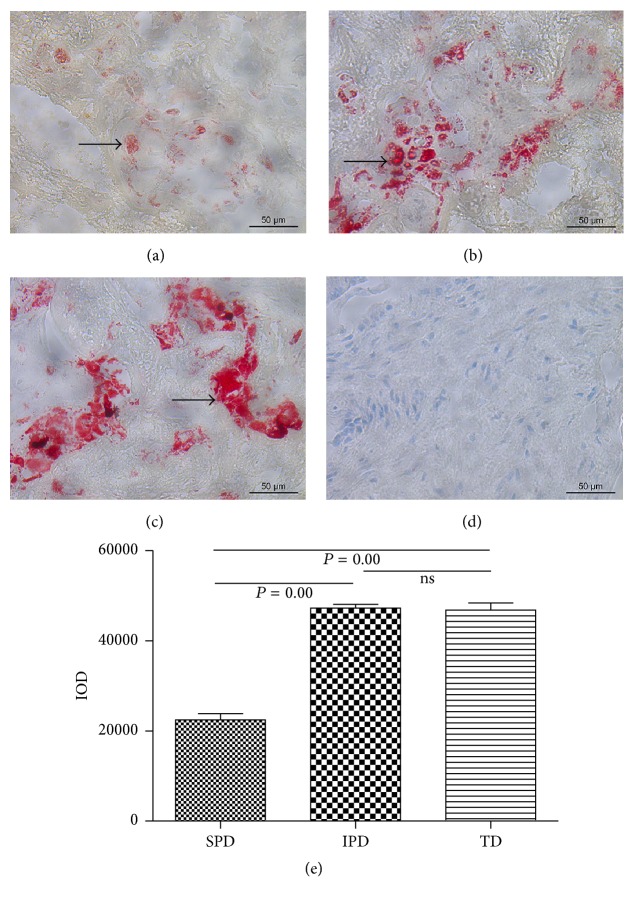
Lipid deposition assessed by Oil Red O staining in placental tissues from women with spontaneous preterm (SPD, (a)), iatrogenic preterm (IPD, (b)), and term (TD, (c)) deliveries; (d) blank control samples. Lipid deposition was observed in IPD and TD but not in SPD or the blank controls. Scale bar = 50 *μ*m; original magnification, ×400. (e) Semiquantitative analysis of the lipid content in the placental tissues. Statistical significance was determined by one-way ANOVA followed by SNK.

Lipid deposits were detected in the placental tissues of IPD (Figure 2(b)) and TD (Figure 2(c)) but not in SPD (Figure 2(a)). No lipid deposits were detected in the blank controls (Figure 2(d)). The placental tissues of SPD had greater levels of lipid contents than IPD and TD, but there was no difference between IPD and TD (Figure 2(e)).

### 3.3. Placental ABCA1 and ABCG1 Protein Localization and Content

**Figure 3:**
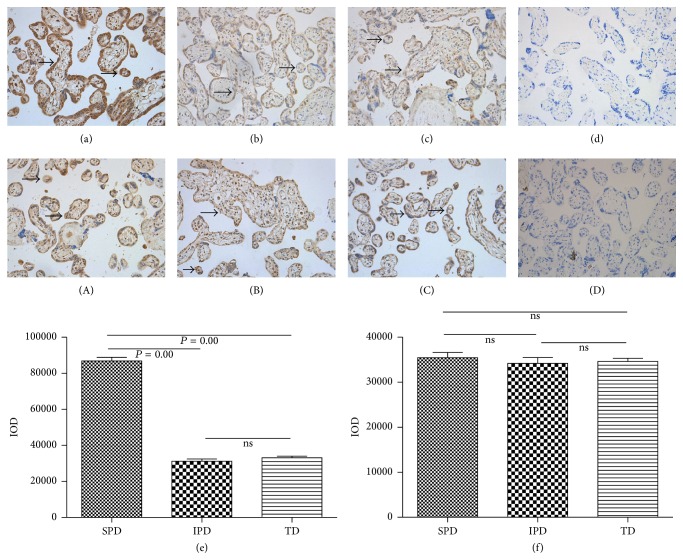
Representative immunohistochemistry images for ABCA1 (a–c) and ABCG1 (A–C) in the placental tissues of women with spontaneous preterm (SPD, (a, A)), induced preterm (IPD, (b, B)), and term (TD, (c, C)) deliveries, with positive staining shown in brown. ABCA1 expression was localized to the syncytium and vascular endothelial cells of the placental tissues, as indicated by the arrowheads. No staining was observed in the isotype controls (d, D). Original magnification, ×200. (e, f) Semiquantitative analysis of ABCA1 (e) and ABCG1 (f) protein expressions. Statistical significance was determined by one-way ANOVA followed by SNK.

ABCA1 (Figures 3(a)–3(c)) and ABCG1 (Figures 3(A)–3(C)) expressions were localized to the syncytium and vascular endothelial cells of the placental tissues. No staining was observed in the isotype controls (Figures 3(d) and 3(D)). SPD had higher levels of placental ABCA1 protein expression than IPD and TD; there was no difference between IPD and TD (Figure 3(e)). In contrast, the ABCG1 protein expressions in the three groups were similar (Figure 3(f)).

### 3.4. Placental ABCA1 and ABCG1 Protein Expressions

**Figure 4:**
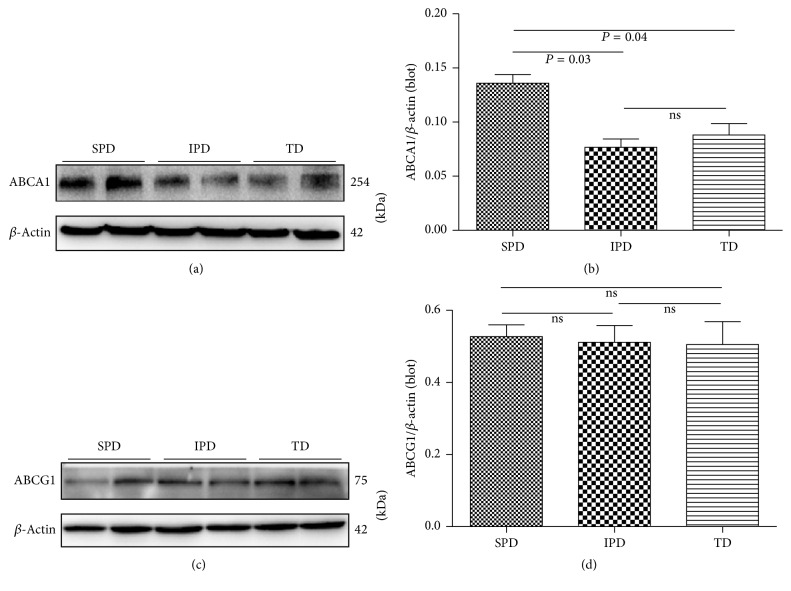
Western blot analysis of placental ABCA1 and ABCG1 in women with spontaneous preterm (SPD), induced preterm (IPD), and term (TD) deliveries. (a, c) Representative Western blot; (b, d) densitometry measurements (mean ± SD) of Western blot bands. Statistical significance was determined by one-way ANOVA followed by SNK.

All samples showed a band at 254 kDa corresponding to the molecular mass of the ABCA1 protein and at 75 kDa corresponding to the molecular mass of the ABCG1 protein (Figures 4(a) and 4(c)). SPD had higher levels of placental ABCA1 protein expression than IPD and TD (Figure 4(b)); there was no difference between IPD and TD (Figure 4(b)). In contrast, there were no differences in the ABCG1 protein expression among the three groups (Figure 4(d)).

### 3.5. Placental* ABCA1* and* ABCG1* Gene Expressions

**Figure 5:**
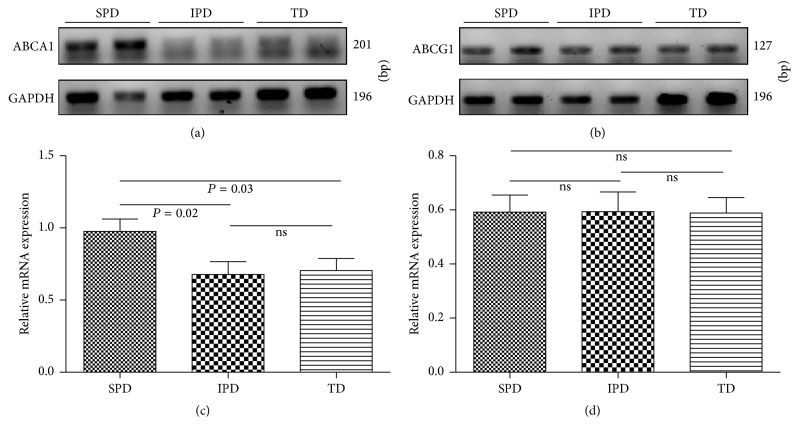
Agarose gel electrophoresis of* ABCA1* (a) and* ABCG1* (b). Placental* ABCA1* (c) and* ABCG1* (d) mRNA expressions in women with spontaneous preterm (SPD), induced preterm (IPD), and term (TD) deliveries. Bars represent mean ± SD. Statistical significance was determined by one-way ANOVA followed by SNK.

SPD had greater levels of placental* ABCA1* mRNA expression than IPD and TD; there was no difference between IPD and TD (Figure 5(c)). The* ABCG1* mRNA expression levels in the three groups were similar (Figure 5(d)).

## 4. Discussion

In our study, we found that the levels of HDL, TG, LDL, and TCHO in the maternal blood were significantly lower in SPD than in IPD and TD. However, HDL, LDL, and TCHO (but not TG) in the cord blood were significantly higher in SPD than in IPD and TD. The extracellular lipids' content in the placenta was significantly lower in SPD than in IPD and TD. We also found that ABCA1 protein and mRNA expressions in the placenta were significantly higher in SPD than in IPD and TD, while the ABCG1 protein and mRNA expressions showed no difference among the three groups. The placenta is an organ of nutrient exchange between mother and fetus during pregnancy. Almost all of the nutrients required for fetal development and maturation can be transported through the placenta. In the human placenta, ABCA1 is mainly expressed in endothelial cells, the surface of the syncytiotrophoblast membrane, and villous cytotrophoblast cells [19, 20]. Christiansen-Weber et al. [28] have shown that ABCA1 has a role in both placental cholesterol homeostasis and fetal-placental cholesterol transport. It has also been reported that the placental ABCA1 protein regulates the flow of maternal-fetal cholesterol, where maternal plasma cholesterol is taken up by placental trophoblastic cells and released into the fetal circulation system at the basal side of the trophoblastic cells [29].

Although the placental ABCA1 transmembrane protein plays a very important role in cholesterol metabolism at the maternal-fetal interface, only recently has attention been paid to the role of this protein in pregnancy-related diseases [19]. Placental ABCA1 expression levels differ in women with different types of pregnancy-related diseases. Chigusa et al. [26] have shown that ABCA1 expression in the placenta of pregnant women with preeclampsia was significantly lower than that of pregnant women without preeclampsia. Albrecht et al. [27] have found that ABCA1 expression in the placenta of pregnant women with primary antiphospholipid syndrome was lower than that of pregnant women with or without preeclampsia. Moreover, the levels of TCHO, TG, and LDL were found to be significantly higher and the levels of HDL were significantly lower in pregnant women with preeclampsia than in pregnant women without preeclampsia. ABCA1 expression negatively correlated with LDL and positively correlated with HDL. Christiansen-Weber et al. [28] have found that knockdown of the* ABCA1* gene in mice resulted in severe embryonic development delays, neonatal death, and fetal loss.

Westerterp et al. [30] have shown that the ABCA1 protein also has anti-inflammatory effects. Knockdown of the* ABCA1* gene in a macrophage cell line resulted in reduced levels of tumor necrosis factor-*α* (TNF-*α*), interleukin-1 (IL-1), and IL-6. ABCA1 and HDL can inhibit the release of inflammatory signals through Toll-like receptors. These receptors play a key role in the process of inflammation in the body, which can inhibit the transcription of liver X receptor target genes by interferon regulatory factor 3. Cheng and Zhang [31] have suggested that immune factors play a key role in the pathogenesis of unexplained noninfectious SPD. An imbalance in cytokine expression involving an increase in TNF-*α*, IL-6, IL-8, and IL-1 expressions and a decrease in IL-10 expression can promote an increase in prostaglandins. This activates cellular immunity leading to uterine smooth muscle contraction and cervical ripening, which can induce SPD. Catov et al. [32] have reported that the ABCA1 protein can also regulate the macrophage-mediated immunological response by transporting IL-1, oxygen free radicals, and other intracellular substances to regulate the expression of inflammatory cytokines and participate in the translocation of phosphatidylcholine, which may play a role in the apoptotic cascade. To date, at least two pathways have been proposed to explain how ABCA1 mediates cholesterol efflux from the cell [33, 34]. In the first pathway, apolipoprotein A-I (apoA-I) binds ABCA1 at the cell surface, where lipids are picked up by apoA-I and then internalized and targeted to late endosomes. In the second, apoA-I forms complexes with phospholipids and cholesterol at the cell surface in a process that is promoted by ABCA1 activity. The apoA-I-lipid complexes are then resecreted from the cell by exocytosis. Azuma et al. [35] have shown that apoA-I is internalized inside the cell and colocalizes with cell surface-derived ABCA1 on endosomal compartments, contributing to HDL formation when excess lipoprotein-derived cholesterol has accumulated in the cells. Two barriers block maternal cholesterol from being transported into the fetal circulation in the placenta: one is villous endothelial cells and the other is villous syncytiotrophoblasts, in which ABCA1 has been described [19, 21]. Based on these findings, our results suggest that the abnormal expression of placental ABCA1 may induce lipid metabolism disorder in the placenta, which leads to the occurrence of SPD, and there may be two mechanisms by which ABCA1 affects the SPD risk. First, abnormal expression of ABCA1 in the placenta dysregulates lipid metabolism in trophoblastic cells, leading to trophoblast cell damage. Second, abnormal expression of ABCA1 in the placenta promotes abnormal immune regulation and cytokine secretion by placental macrophage cells, increasing the risk of SPD.

In conclusion, to our knowledge, this is the first demonstration of an association between abnormal human placental ABCA1 expression and preterm delivery. Our results suggest that abnormal placental expression of ABCA1 may be an important factor in preterm deliveries. However, further research is required to elucidate whether abnormal placental expression of ABCA1 causes SPD.

## Figures and Tables

**Table 1:** Characteristics of the study population (*n* = 145).

	*T* (*n* = 145)	SPD (*n* = 51)	IPD (*n* = 46)	TD (*n* = 48)	*P*_1_	*P*_2_	*P*_3_
Age (years)	27.80 ± 0.70	27.00 ± 0.89	27.50 ± 1.54	28.80 ± 1.15	0.955	0.560	0.736
BMI (kg/m^2^)	22.54 ± 0.26	22.50 ± 0.55	22.39 ± 0.52	22.73 ± 0.27	0.984	0.934	0.861
GD (weeks)	35.44 ± 0.65	32.90 ± 0.69	33.91 ± 0.81	39.50 ± 0.30	0.514	0.000	0.000

GD: gestation of delivery; *T*: total. *P*_1_: SPD versus IPD; *P*_2_: SPD versus TD; *P*_3_: IPD versus TD. Values are mean ± SD. Statistical significance was determined using one-way ANOVA followed by SNK.
